# Self-esteem instability in major depressive disorder: a computational analysis of expectation updating in response to performance feedback

**DOI:** 10.3389/fpsyt.2026.1887088

**Published:** 2026-08-07

**Authors:** Nilay Bilgin, Cemre Candemir, Kaya Oguz, Erkin Can Bilgin, Yigit Erdogan, Mehmet Cagdas Eker, Ali Saffet Gonul

**Affiliations:** 1 SoCAT Lab, School of Medicine, Ege University, Izmir, Türkiye; 2 Department of Psychiatry, Bornova Turkan Ozilhan State Hospital, Izmir, Türkiye; 3 Faculty of Computer sciences, International Computer Institute, Ege University, Izmir, Türkiye; 4 Department of Computer Engineering, Izmir Economy University, Izmir, Türkiye; 5 Department of Psychiatry, School of Medicine, Ege University, Izmir, Türkiye; 6 Faculty of Health Sciences, Department of Neuroscience, Ege University, Izmir, Türkiye

**Keywords:** Major depressive disorder (MDD), negative feedback, performance feedback, positive feedback, self-esteem (Source: MeSH, self-esteem instability

## Abstract

**Introduction:**

Major Depressive Disorder (MDD) is consistently associated with low self-esteem; however, how self-evaluative beliefs are updated in response to environmental feedback in MDD remains poorly understood. In this study, we tested the hypothesis that low self-esteem in MDD reflects dynamic instability driven by altered processing of social-evaluative information, rather than a simple static deficit.

**Methods:**

The study enrolled 60 patients with MDD and 30 healthy control participants. All participants completed a Social Comparison–Based Performance Task in which experimentally manipulated positive and negative performance feedback, presented relative to others, was used to modulate perceived social rank. Trial-by-trial changes in performance expectations following feedback were modeled computationally to derive sensitivity coefficients for positive (k_positive) and negative (k_negative) feedback.

**Results:**

Relative to healthy controls, the MDD group exhibited significantly lower baseline self-esteem, as measured by the Rosenberg Self-Esteem Scale (RSES), as well as lower initial performance expectations at task onset (p < 0.001). Computational modeling revealed that patients with MDD exhibited significantly greater sensitivity of performance expectation updating to both positive (p < 0.001, r = −0.45) and negative (p < 0.001, r = −0.53) feedback compared with healthy controls.

**Limitations:**

Study limitations include the lack of control for psychotropic medications and the recruitment of a sample restricted to outpatients with moderate symptom severity.

**Conclusions:**

Low self-esteem in MDD is better characterized by instability in self-related belief dynamics rather than a simple static deficit. Therapeutic interventions may therefore benefit from targeting heightened sensitivity to environmental feedback and promoting self-esteem stability, rather than focusing solely on increasing self-esteem levels.

## 1 Introduction

Major Depressive Disorder (MDD) has long been associated with low self-esteem in the literature (1–4). Although classical cognitive models emphasize low self-esteem as a vulnerability factor for depression (1), accumulating evidence suggests that instability in self-esteem rather than self-esteem level alone may be a more critical marker of depressive risk (5–7).

The Hierometer Theory conceptualizes self-esteem as a socio-evolutionary gauge that enables individuals to track their relative positions within social hierarchies. Within this framework, self-esteem functions as an internal indicator of perceived social rank, reflecting one’s vertical standing within a hierarchy. Accordingly, individuals with higher self-esteem tend to perceive themselves as occupying a more respected and admired position within the social hierarchy (8). From this perspective, environmental factors that shape social comparison and performance feedback can induce dynamic fluctuations in self-esteem. Such fluctuations—commonly referred to as self-esteem contingency and self-esteem instability—are central to understanding how individuals respond to social-evaluative information (6, 9). Self-esteem instability captures the magnitude of short-term fluctuations in self-esteem over time, whereas self-esteem contingency refers to the extent to which self-esteem varies in response to self-relevant events (10). Crucially, empirical evidence suggests that such instability is deeply rooted in deficits within the internal self-structure, particularly low self-concept clarity and impaired self-representational stability, which leave the individual overly dependent on external validation (11, 12).

According to the cognitive model of depression, the processing of external information exhibits a negative bias, prioritizing the processing of negative information (1, 13). Previous research on feedback processing in depression has emphasized several factors: interpretation and updating biases (14, 15); cognitive inflexibility (16); the devaluation of positive information through the mechanism of “cognitive immunization” against disconfirmatory evidence (17, 18); and notably, a linear decline in optimistic belief-updating that correlates with symptom severity (19, 20). This cognitive process underlies how individuals update their internal schemata based on new experiences. Accordingly, the extent to which self-related beliefs are adjusted depends on the precision of the prior belief, the characteristics of the new information—such as its valence and perceived credibility—and the degree of discrepancy between the prior belief and the new information (21–23). Notwithstanding these advances, the extent to which altered expectation updating contributes to self-esteem instability—particularly when embedded within explicit social-hierarchy-based feedback systems—remains largely unexplored.

Although existing scales measure the contingency of self-esteem on environmental factors (5), their reliance on self-report limits mechanistic interpretability and renders them susceptible to social desirability bias. Moreover, self-report methods cannot adequately capture automatic belief-updating processes. This highlights the need for experimental paradigms that implicitly manipulate social comparison to objectively probe self-esteem dynamics.

This study aimed to examine how positive and negative performance feedback modulates self-esteem dynamics through expectation updating in patients with Major Depressive Disorder (MDD), compared with healthy controls, using a social-hierarchy-based implicit measurement paradigm designed to overcome the limitations of self-report scales. Based on cognitive models of depression, we hypothesized that individuals with MDD would exhibit greater sensitivity to negative than to positive social performance feedback.

## 2 Methods

### 2.1 Participants

A total of 108 participants were recruited, including 71 patients diagnosed with Major Depressive Disorder (MDD) from the Department of Psychiatry at Ege University School of Medicine and 37 healthy controls matched for age, sex, and education, who were enrolled via campus flyers and social media advertisements. Across both groups, inclusion criteria required participants to be between 18 and 60 years of age and to have completed a minimum of 8 years of formal education. Conversely, individuals were excluded from the MDD cohort if they presented with any current or lifetime comorbid psychiatric diagnosis, psychotic symptoms, a clinical presentation severe enough to preclude task participation, or visual impairments affecting performance. Healthy control participants were excluded if they had any current or lifetime history of a psychiatric diagnosis, or presented with visual impairments that could compromise task performance. Participants were enrolled between March 2023 and January 2025. The authors assert that all procedures contributing to this work comply with the ethical standards of the relevant national and institutional committees on human experimentation and with the Helsinki Declaration of 1975, as revised in 2013. All procedures involving human subjects were approved by the Ethics Committee of Ege University (approval number: 23-2.1T/43, approval date: February 23, 2023). Written informed consent was obtained from all participants.

### 2.2 Clinical assessments

Trained psychiatrists (NB and ECB) conducted diagnostic interviews using the Structured Clinical Interview for DSM-5 (SCID-5; First et al., 2015). Clinical interviews and all subsequent assessments were completed within a three-day window to ensure clinical stability relative to the experimental task. Depression severity was assessed in the patient group using the 17-item Hamilton Depression Rating Scale (HAM-D; Hamilton, 1960) and the Beck Depression Inventory (BDI; Beck et al., 1961). Baseline self-esteem was measured in all participants using the Rosenberg Self-Esteem Scale (RSES; Rosenberg, 1965).

### 2.3 Experimental design and procedure

**Figure 1:**
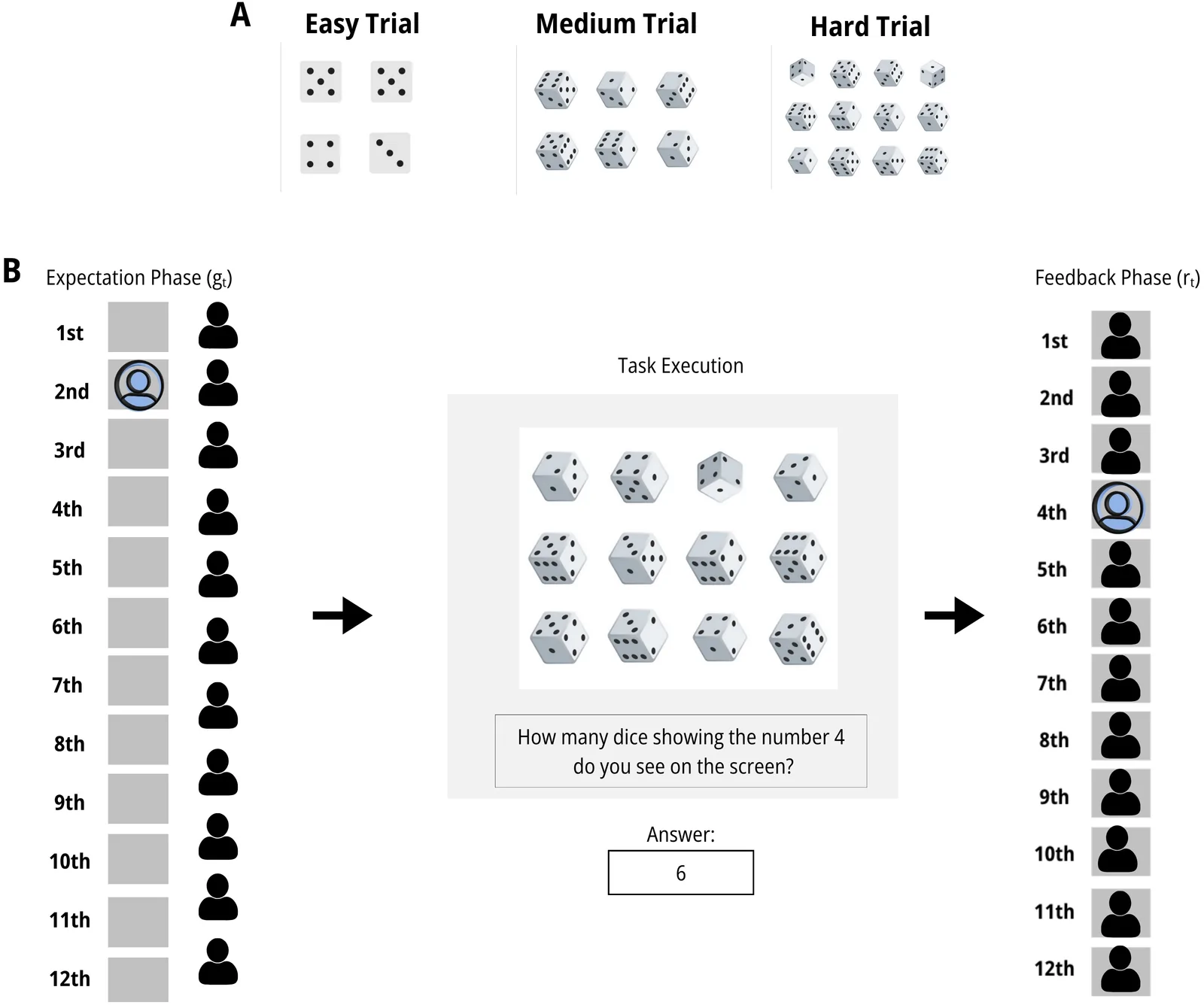
Schematic of the social-comparison-based performance task. **(A)** Task Stimuli and Difficulty Levels: Representation of the dice-counting trials. The task includes three difficulty levels: easy, medium and hard. **(B)** Trial and Block Structure: Schematic representation of the task flow. The experiment consists of 24 blocks in total. Each block begins with participants reporting their rank expectation ( $g_{t}$). This is followed by 6 counting trials where participants answer questions such as “How many dice showing the target number do you see?”. At the end of each block, participants receive manipulated performance feedback ( $r_{t}$).

Participants completed a “Social-Comparison-Based Performance Task” designed to modulate perceptions of social hierarchy (**Figure 1**).

#### 2.3.1 Social-comparison-based performance task

Building on Hierometer Theory (8), we developed a “Social-Comparison-Based Performance Task” in-house specifically for this study (**Figure 1**). One of the authors (K.O.) designed and programmed the software to measure instantaneous reactions to rank-related feedback. Within this task, trial-by-trial rank expectations were conceptualized as latent self-evaluative beliefs, allowing belief updating in response to social feedback to be quantified independently of objective task accuracy.

To reinforce the social comparison effect and highlight hierarchical perception, a vertical ranking scale ranging from 1 to 12 was used in the experimental setting, where rank 1 represented the highest and rank 12 represented the lowest task performance. Participants were informed that they were competing against 11 real opponents with similar demographic characteristics (same gender, education, and ±5 years age range). To enhance credibility, profile photos generated by artificial intelligence representing the 11 virtual opponents, specifically designed to display neutral facial expressions, were displayed on the ranking scale. This arrangement aimed to simulate a genuine social competitive environment.

The task relies on visual attention and calculation skills. Participants were presented with various combinations of dice on a computer screen and were instructed to report the frequency of a specific target number in each trial (e.g., “How many dice showing the number 4 do you see on the screen?”). The task consisted of 24 blocks, each consisting of six trials varying in difficulty (**Figure 1A**). In each trial, the dice stimuli were presented on the screen for a fixed duration of 2.5 seconds. Immediately following stimulus offset, a dedicated response screen appeared, prompting participants to enter their answers. In the easy trials, four dice were displayed in a two-dimensional (2D) view with only a single face visible. The medium trials presented six dice with three faces visible, whereas the hard trials included nine or twelve dice with three faces visible, yielding a three-dimensional (3D) perspective for both conditions.

Before each block, participants were asked to form expectations ( $g_{t}$) of their probable rank within this 12-person hierarchy. At the end of the block, regardless of their actual performance, they were presented with a manipulated performance feedback ( $r_{t}$) (**Figure 1B**). To maintain the perceived authenticity of the social competition, the initial rank was assigned randomly. Subsequent feedback was determined iteratively as $r_{t}=r_{t-1}\pm \text{rand}[0,4]$, allowing for the observation of responses to discrepancies between expected and reported positions. Within this algorithm, the direction of change (positive or negative) was pseudo-randomized rather than being symmetrically distributed across trials. To ensure that the generated feedback remained strictly within the boundaries of the 12-person social hierarchy, boundary constraints were mathematically imposed such that if $r_{t}<1$, it was truncated to 1, and if $r_{t}>12$, it was truncated to 12. This feedback generation was entirely decoupled from and independent of both the participants’ objective task performance and their explicitly stated subjective expectations. Except for these essential hierarchy boundaries, the algorithm imposed no further task-dependent constraints or strategic anchoring on the rank trajectories. Finally, to verify that these randomized rank variations did not compromise task engagement, a formal manipulation check recorded participants’ responses to two explicit credibility questions at the conclusion of the experiment (1): “Did you notice anything unusual during the task?” and (2) “Did you believe your opponents’ performances were in real-time, or did you suspect they were generated by a predetermined algorithm?”

### 2.4 Computational modeling of expectation updating

To quantify how participants updated self-related expectations in response to performance feedback, we implemented a computational belief-updating model grounded in Bayesian inference and active inference frameworks. Within this framework, self-evaluative beliefs are conceptualized as latent expectations that are continuously updated in response to incoming evidence, reflecting the precision-weighted integration of prior beliefs and sensory feedback.

At the beginning of each block t, participants reported their expected social rank within the competitive hierarchy ( $g_{t}$). Following task performance, participants received experimentally manipulated feedback regarding their rank ( $r_{t}$). The discrepancy between expected and observed rank was formalized as a prediction error ( $\delta_{t}=g_{t}-r_{t}$), representing the degree to which incoming feedback violated prior expectations.

Expectation updating was modeled as:


$$g_{t+1}=g_{t}-\beta_{t}$$

where the update magnitude $(\beta_{t}$) reflects the degree to which prediction errors were incorporated into subsequent expectations. In line with Bayesian learning principles, the update magnitude was defined as the product of the prediction error and an individual-specific feedback sensitivity coefficient ( k), which indexes the precision assigned to feedback signals:


$$\beta_{t}=k\times \delta_{t}$$

Higher k values indicate greater precision-weighting of feedback relative to prior beliefs, resulting in larger belief updates, whereas lower values indicate more stable expectations that are less influenced by external input.

To capture asymmetries in belief updating, separate feedback sensitivity parameters were estimated for positive and negative feedback. Positive feedback sensitivity ( $k_{positive}$) was applied when feedback was better than expected ( $\delta_{t}\;$> 0), and negative feedback sensitivity ( $k_{negative}$) was applied when feedback was worse than expected ( $\delta_{t}$ < 0). No update was modeled when expected and received ranks were identical ( $\delta_{t}\;$= 0).

Within an active inference framework, these feedback sensitivity parameters can be interpreted as reflecting the precision of self-related beliefs and their susceptibility to environmental feedback. Elevated sensitivity coefficients indicate heightened belief volatility, operationalized in this study as self-esteem instability. Model parameters were estimated individually for each participant by minimizing the discrepancy between predicted and observed rank expectations across blocks, and the resulting coefficients were used for subsequent group-level statistical analyses.

To further characterize updating asymmetry, we define a within-subject difference score at the individual level:


$$\text{Δ}k=k_{positive}-k_{negative}$$

Here, Δk>0 shows stronger sensitivity to positive than negative feedback, leading to larger expectation updates following positive signals. Conversely, Δk<0 reflects a relatively stronger sensitivity to negative feedback, while Δk~0 while exhibits a balanced updating profile. Thus, the asymmetry in expectation updating can be captured directly, independent of the absolute scale of the coefficients.

To rigorously assess the mathematical identifiability and robustness of the estimated model parameters under the proposed task paradigm, a generative parameter recovery analysis was performed. Synthetic datasets were simulated using known, pre-specified parameter configurations drawn from the empirical distribution of the participant pool. These simulated data were then subjected to the identical optimization and fitting procedures applied to the experimental data. The degree of parameter identifiability was quantified by computing Pearson correlation coefficients ( r) between the ground-truth generating parameters and the newly recovered values.

### 2.5 Statistical analyses

The independent variable was the participant group (MDD vs. Healthy Control), and the dependent variables were the *k* coefficients derived from the computational model ( $k_{mean}$, $k_{positive}$, $k_{negative}$), and updating asymmetry parameters ( Δk).

Normality was assessed using the Shapiro-Wilk test. Sociodemographic variables (age, education, etc.) were compared using the Independent Samples *t*-test. However, *k* coefficients and updating asymmetry parameters ( Δk) were non-normally distributed; therefore, group differences were analyzed using the Mann–Whitney *U* test.

To evaluate clinical significance, Cohen’s *d* was calculated for parametric tests, and rank-biserial correlation coefficients *(r)* for non-parametric tests. Analyses focused on three comparisons (1): Patient vs. Control (Positive) (2), Patient vs. Control (Negative), and (3) Patient vs. Control (All Feedback). Statistical significance was set at *p* < 0.05 (95% CI).

## 3 Results

### 3.1 Sample characteristics and clinical data

Initially, 108 participants (HC = 37, MDD = 71) were recruited. To ensure the validity of the computational parameters, participants were retained only if they validated task credibility across both debriefing questions, with failure on either question leading to automatic exclusion. Consequently, a total of 18 participants (HC = 7, MDD = 11) were subsequently excluded from the primary analyses based on three distinct data-quality constraints (1): Unusual reaction times: 5 healthy controls and 2 patients with MDD were excluded for responding faster than the physiological response threshold (2); Insufficient effort: 8 patients with MDD displayed inappropriate or invariant response patterns, indicating a lack of task engagement. Specifically, insufficient effort was operationally defined as failing both of the baseline easy trials within a block and/or entering the exact same numerical response for all six consecutive trials within a single block, suggesting automated or unengaged key-pressing behavior; and (3) Task skepticism: 2 healthy controls and 1 patient with MDD explicitly reported strong skepticism regarding the real-time authenticity of the virtual opponents during the manipulation check. Final analyses were conducted on the remaining sample of 90 participants (HC = 30, MDD = 60).

There were no significant differences between groups regarding age, gender, or education duration (**Table 1**).

**Table 1:** Demographic and clinical characteristics of MDD and control groups.

Variable	MDD (N = 60)	Controls (N = 30)	Statistics
Age, mean years (SD)	37.3 (10.8)	39.9 (12.7)	*t* = -0.96 *df* = 88 *P* = 0.34
Sex, n (%)			*X2* = 0.00
Male	12 (20%)	6 (20%)	*df* = 1
Female	48 (80%)	24 (80%)	*P* > 0.99
Education, median years (SD)	12.8 (3.1)	13.5 (3.3)	*t* = -0.93 *df* = 88 *P =* 0.35
Current medication, n (%)			
Antidepressant (Total)	60 (100%)	NA	NA
SSRIs	38 (63.3%)	NA	NA
SNRIs	19 (31.7%)	NA	NA
NDRI	9 (15.0%)	NA	NA
Tricyclics	1 (1.7%)	NA	NA
HAMD Total Score, mean (SD)	16.6 (3.8)	NA	NA
BDI Total Score, mean (SD)	24.1 (4.4)	NA	NA

MDD, Major Depressive Disorder; N, Sample size; SD, Standard Deviation; HAM-D, Hamilton Depression Rating Scale; BDI, Beck Depression Inventory; SSRI, Selective Serotonin Reuptake Inhibitor; SNRI, Serotonin-Norepinephrine Reuptake Inhibitor; NDRI, Norepinephrine-Dopamine Reuptake Inhibitor; *NA, Not Applicable*.

### 3.2 Baseline characteristics and experimental task metrics

Pre-task measurements showed that the MDD group had lower Rosenberg Self-Esteem Scale (RSES) scores compared to controls (*t* (88) = -15.185, *p* < 0.001). Similarly, initial performance expectations ( $g_{t=0}$) in the social-comparison-based performance task were lower in MDD patients (*p* < 0.001). Additionally, no differences were found in correct response counts (*p* = 0.18) or reaction times (*p* = 0.25) during the task (**Table 2**).

**Table 2:** Baseline self-esteem, initial performance expectations, and task performance metrics.

Variable	MDD (N = 60)	Controls (N = 30)	Statistics
RSES Total Score, mean (SD)	13.5 (5.4)	26.5 (2.7)	*t* = -15.18 *df* = 88 *P* = < 0.001
Baseline Performance Expectation (g_t=0_), mean (SD)	3.9 (1.2)	2.0 (0.6)	*t* = -7.53 *df* = 88 *P* = < 0.001
Number of Correct Responses Per Block, mean (SD)	4.1 (1.2)	4.5 (0.9)	*t* = 1.35 *df* = 88 *P* = 0.18
Reaction Time Per Block, mean (SD) (ms)	1213.3 (540.0)	1056.7 (440.0)	*t* = -1.21 *df* = 88 *P* = 0.25

RSES, Rosenberg Self-Esteem Scale; g(t=0), Baseline rank expectation; ms, Milliseconds; SD, Standard Deviation.

### 3.3 Computational parameters of feedback sensitivity

**Figure 2:**
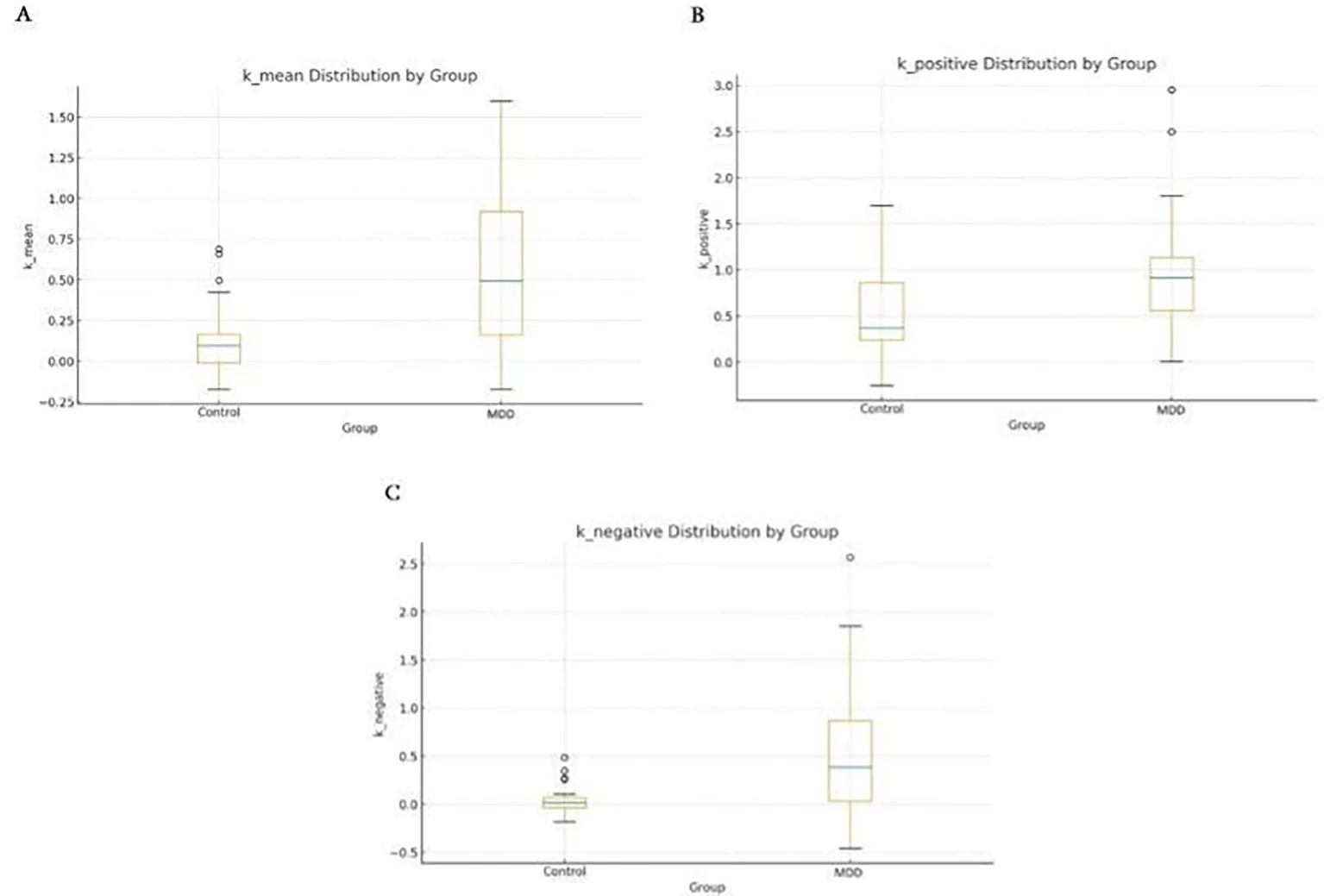
Comparison of feedback sensitivities between groups. **(A)** Mean Feedback Sensitivity ( $k_{mean}$) for Control and MDD groups. **(B)** Positive Feedback Sensitivity ( $k_{positive}$) for Control and MDD groups. **(C)** Negative Feedback Sensitivity ( $k_{negative}$) for Control and MDD groups.

Regardless of feedback direction, the sensitivity coefficient ( $k_{mean}$) at which MDD patients updated their performance predictions based on feedback was significantly higher than that of healthy controls (*U* = 1435.0, *p* = 4.76 × 10^-6^). The large effect size (*r* = -0.594) indicates that the patient group was markedly more influenced by feedback (**Figure 2A**).

When receiving positive feedback better than their prediction, the MDD group exhibited a significantly higher coefficient for updating performance expectations compared to controls (*U* = 1096.5, *p* = 0.0009). The effect size (*r* = -0.450) denotes a medium-to-large effect (**Figure 2B**).

The MDD group also demonstrated a significantly higher update coefficient compared to controls when receiving negative feedback worse than expected (*U* = 1374.5, *p* = 4.97 × 10^-5^). The effect size (*r* = -0.527) indicates a large difference (**Figure 2C**).

**Figure 3:**
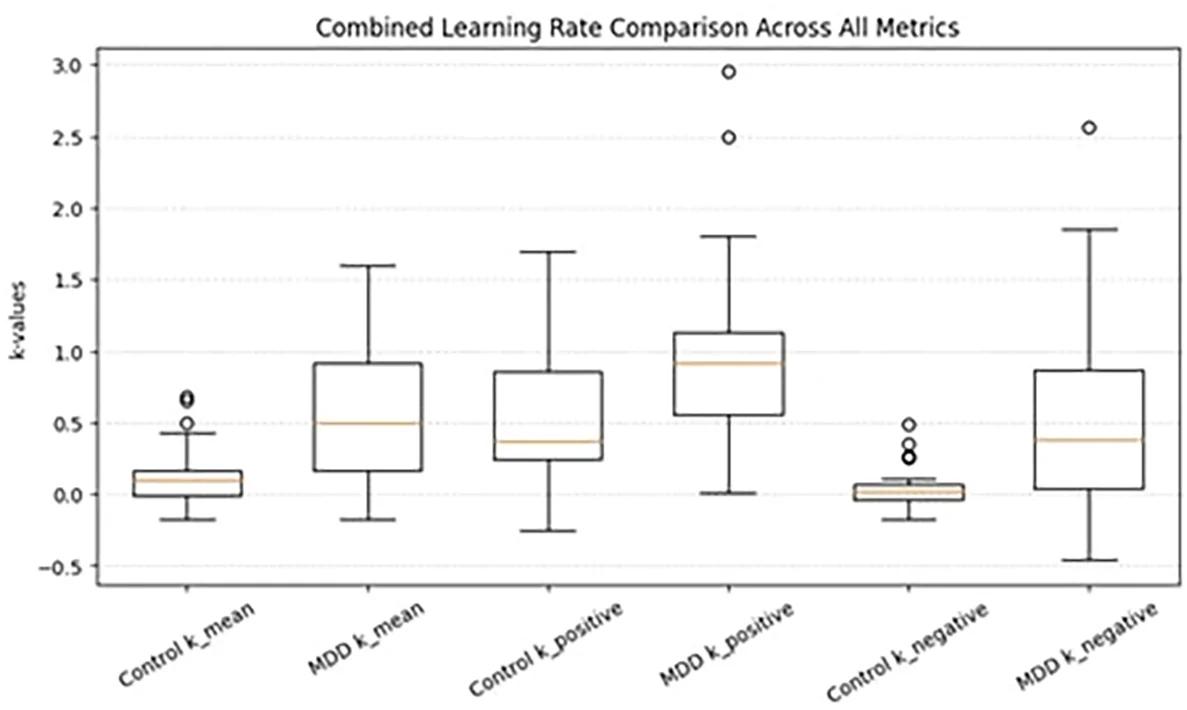
Combined visualization of all sensitivity (k) parameters.

Examination of the within-group distribution revealed that $k_{positive}$ values in the MDD group showed a right-skewed distribution, with some patients developing extreme sensitivity (outliers) to feedback. While significant group differences were observed for both feedback types, the effect size for $k_{negative}$ (*r* = -0.53) was stronger than for $k_{positive}$ (*r* = -0.45) (**Figure 3**).

Regarding the asymmetry of expectation updating, both groups exhibited positive median *Δk* values, reflecting an overall dominance of learning from positive feedback. A Mann–Whitney U test revealed a significant group difference in the *Δk* parameters (*p* < 0.001, *r* ≈ –0.50).

**Figure 4:**
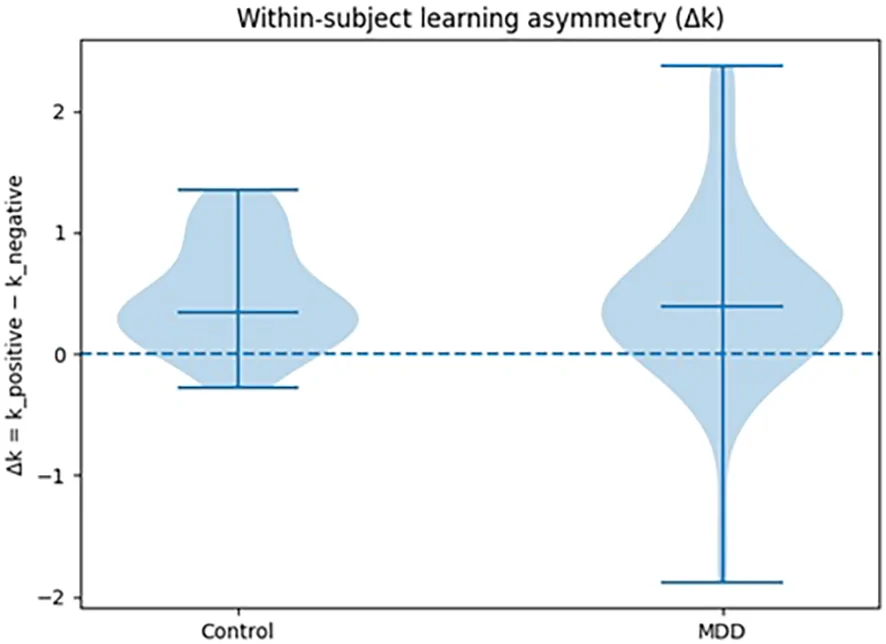
Distribution of within-subject expectation updating asymmetry (*Δk* = $k_{positive}$– $k_{negative}$) in healthy controls and MDD participants.

As visualized in the violin plots in **Figure 4**, the healthy control group demonstrated a narrow distribution of values concentrated above zero. In contrast, the MDD group was characterized by substantially greater dispersion and a pronounced negative tail. While $k_{positive}$ values in the MDD group showed a right-skewed distribution with several outliers, $k_{negative}$ exhibited the largest group effect size compared to the control group.

#### 3.3.1 Model validation and parameter recovery

**Figure 5:**
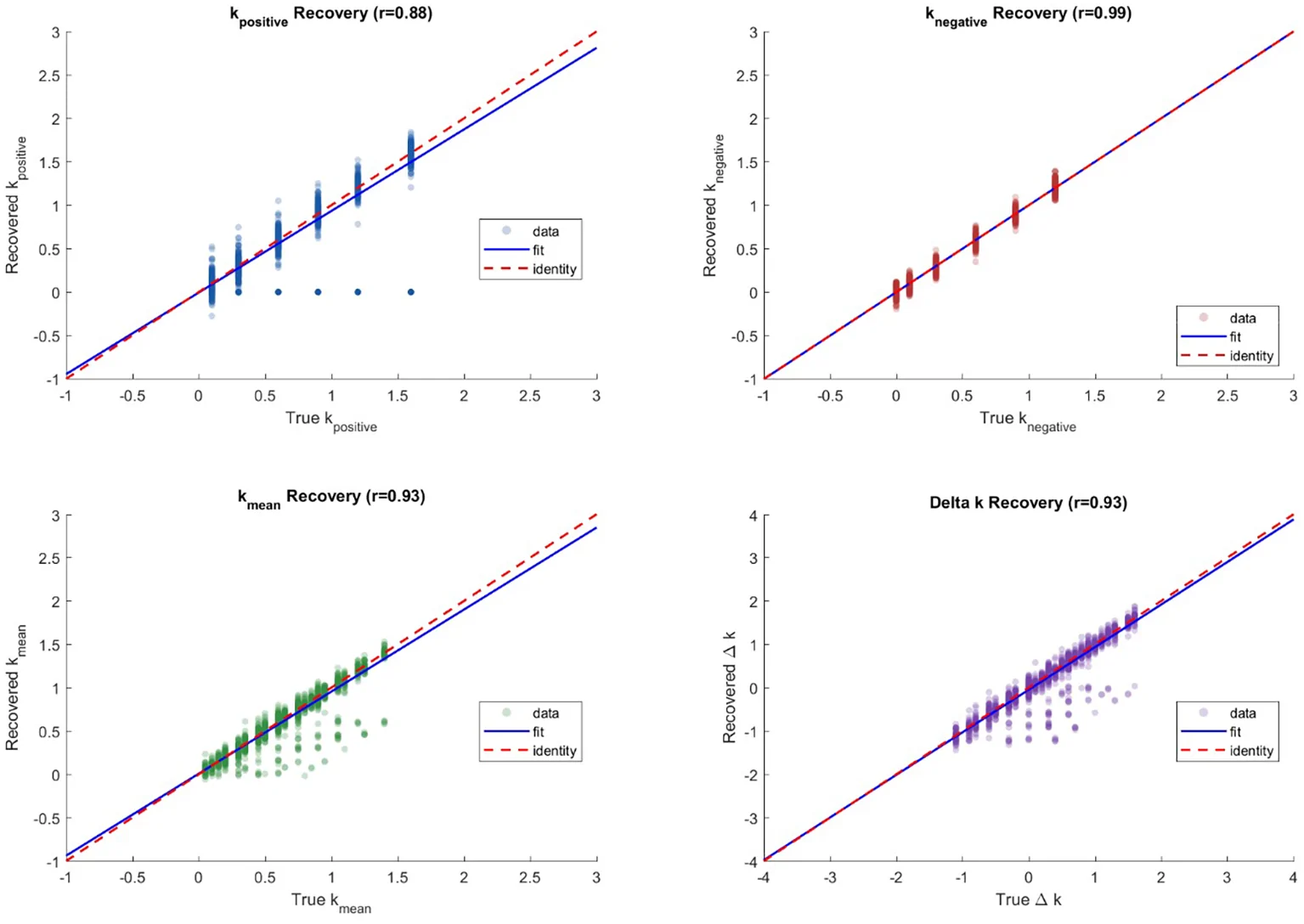
Generative parameter recovery analysis. Scatter plots illustrating the correlations between the ground-truth parameters (x-axis) and the computationally recovered parameters (y-axis) for ( $k_{\text{positive}}$), ( $k_{\text{negative}}$), ( $k_{\text{mean}}$), and ( Δk). The dashed red line represents the diagonal identity line ( y=x), and the solid blue line represents the linear regression line.

The parameter recovery analysis demonstrated excellent identifiability and robustness across all estimated coefficients (**Figure 5**). Pearson correlation coefficients (*r*) revealed an exceptionally strong correspondence between the true parameters and recovered values for negative learning sensitivity ( $k_{\text{negative}}$: *r* = 0.99), overall sensitivity ( $k_{\text{mean}}$: *r* = 0.92), and the learning asymmetry parameter ( Δk: *r* = 0.93). The recovery of positive learning sensitivity ( $k_{\text{positive}}$) was also robust (*r* = 0.88), despite demonstrating marginally higher baseline variability at the lower end of the parameter range. Across all parameters, the estimated values closely aligned with the diagonal identity line, indicating that the task structure and optimization procedure provide sufficient sensitivity to reliably isolate individual differences in belief-updating dynamics without algorithmic confounding.

### 3.4 Independence of computational parameters from acute clinical severity and baseline self-esteem

Exploratory correlation analyses revealed no statistically significant relationships between any of the computational parameters ( $k_{\text{mean}}$, $k_{\text{positive}}$, $k_{\text{negative}}$, Δk) and clinical severity scores (HAM-D, BDI) or baseline self-esteem (RSES) within the MDD group (all *p* >.05; see **Supplementary Section 1**; **Supplementary Tables 1, 2** for detailed statistical reporting).

## 4 Discussion

This study examined how self-esteem dynamics in MDD respond to environmental feedback within a social hierarchical context. Extending beyond our initial hypothesis, we demonstrated that individuals with MDD exhibit significantly heightened sensitivity to both positive and negative feedback. This pattern indicates that the core cognitive alteration in MDD is characterized by a generalized instability in self-evaluative belief updating rather than a static deficit in self-esteem level alone.

Baseline self-esteem levels and initial performance expectations ( $g_{t=0}$) were significantly lower in the MDD group, consistent with established literature (1–4). Viewing these low baseline expectations through the lens of Hierometer Theory—which conceptualizes self-esteem as a status-tracking gauge—suggests that depressed individuals perceive their social rank as fundamentally disadvantageous even prior to external evaluation. This pre-existing state of low expectations aligns with evidence that perceived low status directly drives diminished self-esteem and depressive affect (24), pointing to a baseline disruption in social status-monitoring mechanisms.

Group comparisons revealed that the MDD group displayed significantly higher sensitivity coefficients across both valences compared to healthy controls. While within-group analyses indicated that the median sensitivity to positive feedback remained higher than to negative feedback in the MDD cohort, the most substantial statistical divergence from healthy controls occurred in the $k_{\text{negative}}$ parameter. This suggests that while individuals with MDD exhibit clinical heterogeneity in processing positive evaluation, they demonstrate a highly consistent, pervasive hypersensitivity to negative feedback. Distributional analyses further underscored this asymmetry; while healthy controls maintained a narrow, stable clustering of their update bias, the MDD group displayed much broader variability characterized by a pronounced “negative tail.” Crucially, capturing such nuanced distributional variations and their underlying mechanistic drivers necessitates advanced computational approaches. Importantly, while previous computational models captured social estimation patterns using larger samples, they lacked validation in MDD (25, 26). Our findings demonstrate that clinical self-esteem instability in MDD is uniquely driven by a pathologic hyper-reactivity to prediction errors across both valences, rather than being a static consequence of rigid prior expectations alone.

Accumulating empirical literature demonstrates that the direction and magnitude of external performance feedback serve as the primary drivers of expectational updates, operating independently of baseline inter-individual differences (27). Because the lower baseline expectations of the MDD group inherently alter the trajectory of prediction errors and feedback streams relative to healthy controls, the observed group differences in k parameters could potentially be viewed as a mathematical artifact of the task structure. Crucially, however, our generative parameter recovery analysis confirmed that the model parameters (specifically $k_{\text{negative}}$ and Δk) could be successfully identified independent of one another under the proposed task architecture. This methodological validation ensures that our findings reflect genuine, stable mechanistic features of belief-updating dynamics in depression rather than an algorithmic confound.

Within this computational framework, the elevated k parameter directly indexes heightened belief volatility, mapping onto the clinical construct of self-esteem instability. This generalized instability can be mechanistically understood through the cognitive architecture of low self-concept clarity and impaired self-representational stability. Specifically, low self-concept clarity—characterized by poorly defined, malleable self-beliefs—leaves individuals with MDD without a stable baseline reference point. Lacking this representational solidity, their computational system becomes hyper-reactive to external evaluative signals, pathologically over-weighting prediction errors ( $k\times \delta_{t}$). Unlike healthy controls, whose resilient self-schema shields against environmental noise, individuals with MDD lack consolidated self-representations to maintain equilibrium. Consequently, external feedback triggers bidirectional volatility: negative feedback induces a severe deflation of self-evaluation ( $k_{\text{negative}}$), while positive feedback prompts abrupt, unmitigated surges in expectations (indexed by $k_{\text{positive}}$).

Intriguingly, the MDD group increased their performance expectations following positive feedback, exhibiting higher overall feedback sensitivity compared to controls. This finding diverges from literature suggesting blunted cognitive responsiveness or cognitive immunization against positive data in depression (17, 18), suggesting that in explicit hierarchical settings, positive feedback can transiently pierce the depressive schema. Conversely, when facing negative feedback, individuals with MDD consistently and drastically lowered their expectations. This reinforces a shared core reactivity to negative evaluation that may be driven by a global tendency toward self-verification and schematic preservation. Because individuals with MDD hold low baseline expectations, negative feedback is highly self-congruent, which drives its rapid integration into established self-schemata to maintain cognitive coherence (28).

In contrast, the resistance of healthy controls to negative feedback is attributable to well-documented “positive illusions,” including illusions of superiority, control, and unrealistic optimism (29–33). Recent computational formulations indicate that an optimistic update bias serves an adaptive, protective function for mental health (34–36), wherein healthy individuals leverage their pre-existing self-concept as a reference point to shield against self-discrepant data (37, 38). Within our paradigm, this self-preservation mechanism is captured by an attenuated negative sensitivity parameter ( $k_{\text{negative}}$), which essentially filters out negative information to maintain self-esteem stability by enabling individuals to contextualize external feedback as merely transient and situational.

Finally, the absence of group differences in reaction times and task accuracy suggests that both cohorts exhibited similar baseline cognitive performance, diverging from reports of severe executive dysfunctions in depression (39–41). This discrepancy is likely attributable to the moderate symptom severity and absence of psychotic features in our sample, as cognitive dysfunction is typically pronounced in specific sub-biotypes and correlates linearly with clinical severity (42–45).

### 4.1 Clinical implications

Our findings suggest that MDD treatment should not solely focus on raising baseline self-esteem levels but must target the regulation of hypersensitivity to external stimuli to promote self-esteem stability. Psychotherapeutic interventions should train patients to contextualize feedback as specific task outcomes rather than global reflections of personal inadequacy.

### 4.2 Limitations

Several limitations should be noted. First, given that the entire MDD cohort was receiving concomitant psychotropic medication, we could not control for potential pharmacological effects on feedback sensitivity. Second, the sample consisted exclusively of outpatients with moderate symptom severity, as evidenced by their HAM-D and BDI scores. Furthermore, the exclusion of patients presenting with severe cognitive impairment, psychotic features, or high suicide risk limits the applicability of our results to more severe clinical presentations. Finally, our explicit social-hierarchy paradigm does not account for broader cognitive variables, such as prior belief precision or the mathematical magnitude of belief-schema discrepancies. This omission limits our ability to mechanistically parse these multifaceted processes within the present sample.

## 5 Conclusion

This study demonstrates that self-esteem in MDD is best characterized by instability in response to social-evaluative feedback, rather than by a static deficit alone. Using an implicit social-hierarchy paradigm and computational modeling, the current study shows that depressed individuals exhibit heightened belief updating to both positive and negative feedback. These findings underscore the importance of targeting self-esteem stability in depression treatment and highlight the value of implicit, computational approaches for elucidating self-related belief dynamics in psychiatric disorders.

## Data Availability

The original contributions presented in the study are included in the article/**Supplementary Material**. Further inquiries can be directed to the corresponding author/s.
