# Supplementary material for: Self-esteem instability in major depressive disorder: a computational analysis of expectation updating in response to performance feedback
Source: Front Psychiatry. 2026 Aug 7;17:1887088. doi: 10.3389/fpsyt.2026.1887088 (PMC13495575; doi:10.3389/fpsyt.2026.1887088)
Supplement: Supplementary file 1 [file Table1.docx]

## Supplementary Section 1: Exploratory Clinical Correlation Analyses within the MDD Cohort

### 1. Rationale and Methodology

To rigorously evaluate whether individual variations in the computationally derived belief-updating parameters scaled with clinical symptom severity or baseline self-esteem within the patient population, exploratory correlation analyses were conducted exclusively within the Major Depressive Disorder (MDD) cohort ($N=60$).

Associations were assessed between all four computational parameters ($k_{\text{mean}}$, $k_{\text{positive}}$, $k_{\text{negative}}$, and $\Delta k$) and three established clinical metrics:

1. The 17-item Hamilton Depression Rating Scale (HAM-D)
2. The Beck Depression Inventory (BDI)
3. The Rosenberg Self-Esteem Scale (RSES)

Given that the computational parameters and clinical scales did not meet the assumptions of normality, Spearman’s rank correlation coefficient ($\rho$) was utilized as the primary distribution-free statistical approach. To ensure statistical robustness and evaluate potential linear relationships, Pearson correlation coefficients ($r$) were also computed as a supplementary sensitivity analysis. The significance threshold was set a priori at $\alpha=0.05$.

### 2. Supplementary Results

As detailed in Table S1 and Table S2, no statistically significant associations were observed between any of the computational learning parameters and clinical metrics within the MDD sample (all $p>0.05$).

The largest, though statistically non-significant, association was observed between the learning asymmetry parameter ($\Delta k$) and baseline self-esteem scores (RSES) ($\rho=0.163$, $p=0.230$). Pearson correlation analyses yielded an identical pattern of findings, confirming the absence of significant linear associations.

Crucially, while the MDD and Healthy Control groups exhibit robust, categorical differences in their baseline computational parameters, these parameters do not fluctuate as a function of current symptom severity or self-esteem levels within the depressed cohort itself. This pattern indicates that the observed computational alterations function as state-independent, invariant trait markers of Major Depressive Disorder rather than temporary correlates of acute clinical severity.

### Table S1. Primary Analysis: Spearman’s Rank Correlations ($\boldsymbol{\rho}$) within the MDD Group

| Parameter | Clinical Scale | Spearman ρ | p-value |
| --- | --- | --- | --- |
| k_mean | HAM-D | 0.107 | 0.418 |
|  | BDI | -0.012 | 0.925 |
|  | RSES | 0.069 | 0.598 |
| k_positive | HAM-D | 0.111 | 0.414 |
|  | BDI | -0.014 | 0.918 |
|  | RSES | 0.151 | 0.266 |
| k_negative | HAM-D | 0.050 | 0.706 |
|  | BDI | -0.047 | 0.722 |
|  | RSES | -0.019 | 0.887 |
| Δk | HAM-D | 0.015 | 0.913 |
|  | BDI | 0.008 | 0.953 |
|  | RSES | 0.163 | 0.230 |

Note: $N=60$. $k_{\text{mean}}$: overall feedback sensitivity; $k_{\text{positive}}$: positive feedback sensitivity; $k_{\text{negative}}$: negative feedback sensitivity; $\Delta k$: learning asymmetry ($k_{\text{positive}}-k_{\text{negative}}$). Significance level set at $p<0.05$.

### Table S2. Supplementary Analysis: Pearson Linear Correlations ($\boldsymbol{r}$) within the MDD Group

| Parameter | HAM-D | BDI | RSES |
| --- | --- | --- | --- |
| k_mean | 0.070 (p=0.593) | -0.061 (p=0.644) | 0.083 (p=0.531) |
| k_positive | 0.090 (p=0.509) | 0.068 (p=0.618) | 0.057 (p=0.675) |
| k_negative | 0.039 (p=0.769) | -0.062 (p=0.638) | -0.044 (p=0.740) |
| Δk | 0.026 (p=0.847) | 0.100 (p=0.464) | 0.118 (p=0.387) |

Note: Values represent Pearson's $r$ with corresponding $p$-values in parentheses. Significance level set at $p<0.05$.
